# Supplementary material for: Prevalence of malaria and helminth infections in rural communities in northern Sierra Leone, a baseline study to inform Ebola vaccine study protocols
Source: PLoS One. 2022 Jul 6;17(7):e0270968. doi: 10.1371/journal.pone.0270968 (PMC9258822; doi:10.1371/journal.pone.0270968)
Supplement: Attachment — Submitted filename: Response to reviewer comments-FINAL.docx [file pone.0270968.s003.docx]

**Reviewers’ comments and actions taken.**

We are grateful to the reviewers for their compliments and critical review of the manuscript. We have found their comments to be useful and we have accordingly revised the manuscript.

Below is a point-by-point account of how we have used the comments to revise the manuscript.

**Comment 1**
1. MDA regimens: lines 117 - 121 indicate that, since 2018, school-aged children (SAC) were the sole recipients of (annual) ivermectin plus albendazole, although this age-group had been receiving albendazole every 6 months up until 2016 when it was deemed no longer necessary.

Can the authors clarify from when SAC received albendazole every 6 months, and indicate how that treatment was integrated with the combined ivermectin-albendazole MDA given to all aged >5 up until 2017. Also, in the Sierra Leonean context, for clarity they should specify what age-group constitutes SAC.

**Response 1**

We have revised the manuscript in lines 122-128 with a clarification of this section as follows

Deworming of School-Aged Children (SAC) with albendazole has been part of the public health system in Sierra Leone for many years. The program defined SAC to be children aged between 5 and 14 years. A national impact survey in 2016 however demonstrated that the intervention was no longer justified. MDA-LF with ivermectin and with albendazole was undertaken annually from 2008-2017 for everyone over 5 years of age. Since 2018 however, this activity has been targeted at only SAC. Deworming of pre-SAC with albendazole continues to be undertaken every six months. This is done through local health facilities and community outreach services.

*.*

**Comment 2**
2. Sample procedures: lines 153 - 159 outline the quality control procedure used for microscopical diagnosis of plasmodial parasites. Was no such confirmatory procedure adopted for the Kato-Katz-based diagnoses?

**Response 2**

We have revised the manuscript in lines 177-179 to improve clarity as follows:

As part of quality control for the procedure, the microscopic examination took place within 30 minutes after sample processing. Ten percent of negative samples and all positive samples were reread by a senior laboratory staff immediately after the first reading to validate the results.

**Comment 3**

3. The rate of agreement to participate by household heads was appreciably lower in the non-riverine community. If there were consistent reasons found for this, they should be detailed.

**Response 3**

We agree to the suggestion and have revised in lines 307-311 to include the following

Response to participation in the survey was better in the riverine than non-riverine communities. The riverine communities are closer to the offices of the Ebola vaccine project. It is conceivable that community engagements activities in the Ebola vaccine project may have impacted positively on the willingness of people in the riverine communities to participate in the survey compared to people in the non-riverine communities.

**Comment 4**

4. The authors should clarify whether the Ebola vaccines under study are intended to be tested in specific age groups.

**Response 4**

We have revised the manuscript in lines 86-87 as follows

The Ebola vaccines are evaluated in persons aged one year and older.

Reviewer #1:

**Comment 5**

The authors present their manuscript on the prevalence of malaria and helminth infections in rural communities in northern Sierra Leone. This is a baseline study to inform Ebola vaccine study protocols. The results are great to present in the manuscript; however, the presentation of the results could be improved to bring clarity to the manuscript. The following are my specific comments:
Abstract section: In the results section of the abstract, the authors could include at least brief results of the lymphatic filariasis and onchocerciasis results.

**Response 5**

We agree with the reviewer. Within the constraints of word limitation, we have included the following statement in the *Results* section (lines 58-59) of the abstract

*Onchocerca volvulus* and *Wuchereria bancrofti* infections were found in 3.3% and 0.4% of participants respectively.

**Comments 6**

Results section
- The headings in the results section should be improved to bring understanding to what is being presented. The first item presented is demography stuff so the authors could have a heading like “Demographic characteristics of the study population”. Currently there is no heading for this presentation. Again such headings as “Helminths” does not tell much. It could have been “Helminth infections in the population” etc

It would have been great if the authors present the different parasites differently under different groups. For instance, the presentation could have been done under the following: Soil Transmitted helminths, Schistosomiasis, blood associated parasites (Onchocerciasis and Wuchereria bancrofti). This would have brought clarity to the results.

**Response 6**

We appreciate the point made by the reviewer. We are however further guided by the “Instructions to Authors” provided by PLOS to keep headings and sub-headings to a minimum.

Taking the reviewer’s suggestions into account and recognizing the limitations imposed by the journal’s requirements, we have revised the manuscript to includes the following sub-headings:

- Soil-transmitted helminths and schistosome infections (line 265)
- Blood associated parasites (*O. volvulus* and *W. bancrofti*) (line 292)

We believe this has improved the clarity of presentation of the results.

**Comment 7**

Under Malaria parasitaemia, the authors could present the co-infection parasites eg how many patients were co-infected with Plasmodium falciparum and P. vivax and all the other coinfections too. The scientific world will be interested to know which parasites infected the people as coinfection.

**Response 7**

The manuscript has been revised in lines 241-247 to include this information as follows.

A single malaria parasite species was identified in 288 slides, two species were identified in 13 slides (11 involved mixed infections of *P. falciparum* and *P. malariae* while two involved mixed infections of *P. falciparum* and *P. ovale*) and speciation was not reported for 8 slides.

**Comment 8**

Also with the helminth infections, the authors do not state which parasites were the co-infections. Such information are important to publish.

**Response 8**

This information is provided in the manuscript (lines 267-271) as follows

The positive helminth infections comprised 102 (12.5%) cases of hookworm, 11 (1.3%) cases of *T. trichiura,* 10 (1.2%) cases of *S. mansoni* and two (0.2%) cases of *A. lumbricoides*. The three stool samples showing mixed infections were two samples with hookworm and *T. trichiura* mixed infection, and one with hookworm and schistosomiasis mixed infection (**Table 2**).

When submitting your revision, we need you to address these additional requirements.

1. Please ensure that your manuscript meets PLOS ONE's style requirements, including those for file naming. The PLOS ONE style templates can be found at 
[https://journals.plos.org/plosone/s/file?id=wjVg/PLOSOne_formatting_sample_main_body.pdf](https://protect-eu.mimecast.com/s/OFqrCqZ14tXjy0kfQukJn?domain=journals.plos.org) and 
[https://journals.plos.org/plosone/s/file?id=ba62/PLOSOne_formatting_sample_title_authors_affiliations.pdf](https://protect-eu.mimecast.com/s/QyX4Cr914S2lRVnsyRkMP?domain=journals.plos.org)

2. Please include a complete copy of PLOS’ questionnaire on inclusivity in global research in your revised manuscript. Our policy for research in this area aims to improve transparency in the reporting of research performed outside of researchers’ own country or community. The policy applies to researchers who have travelled to a different country to conduct research, research with Indigenous populations or their lands, and research on cultural artefacts. The questionnaire can also be requested at the journal’s discretion for any other submissions, even if these conditions are not met.  Please find more information on the policy and a link to download a blank copy of the questionnaire here: [https://journals.plos.org/plosone/s/best-practices-in-research-reporting](https://protect-eu.mimecast.com/s/QT6uCvZ14tAznNLfoXLLj?domain=journals.plos.org). Please upload a completed version of your questionnaire as Supporting Information when you resubmit your manuscript.

3. We note that the grant information you provided in the ‘Funding Information’ and ‘Financial Disclosure’ sections do not match.

When you resubmit, please ensure that you provide the correct grant numbers for the awards you received for your study in the ‘Funding Information’ section

Please review your reference list to ensure that it is complete and correct. If you have cited papers that have been retracted, please include the rationale for doing so in the manuscript text, or remove these references and replace them with relevant current references. Any changes to the reference list should be mentioned in the rebuttal letter that accompanies your revised manuscript. If you need to cite a retracted article, indicate the article’s retracted status in the References list and also include a citation and full reference for the retraction notice.
